# Trends in Life Expectancy and Its Association with Economic Factors in the Belt and Road Countries—Evidence from 2000–2014

**DOI:** 10.3390/ijerph15122890

**Published:** 2018-12-17

**Authors:** Ruhai Bai, Junxiang Wei, Ruopeng An, Yan Li, Laura Collett, Shaonong Dang, Wanyue Dong, Duolao Wang, Zeping Fang, Yaling Zhao, Youfa Wang

**Affiliations:** 1 Global Health Institute, Xi’an Jiaotong University Health Science Center, Xi’an, Shaanxi 710061, China; bairuhai@stu.xjtu.edu.cn (R.B.); junxiang.wei@mail.xjtu.edu.cn (J.W.); fangzp@stu.xjtu.edu.cn (Z.F.); yaling-zhao@163.com (Y.Z.); 2 School of Public Health, Xi’an Jiaotong University Health Science Center, Xi’an, Shaanxi 710061, China; tjdshn@mail.xjtu.edu.cn (S.D.); wanyuedong@foxmail.com (W.D.); 3 Department of Kinesiology and Community Health, College of Applied Health Sciences, University of Illinois at Urbana-Champaign, Illinois, IL 61820, USA; ran5@illinois.edu; 4 Center for Health Innovation, The New York Academy of Medicine, New York, NY 10029, USA; yanliacademic@gmail.com; 5 Department of Population Health Science and Policy, Icahn School of Medicine at Mount Sinai, New York, NY 10029, USA; 6 Department of Clinical Sciences, Liverpool School of Tropical Medicine, Liverpool L3 5QA, UK; laura.collett@bristol.ac.uk (L.C.); Duolao.Wang@lstmed.ac.uk (D.W.)

**Keywords:** Belt and Road, life expectancy, quantile mixed model

## Abstract

In 2013, China launched the Belt and Road (B&R) Initiative in an effort to promote trade and economic collaboration. This study examined the change in life expectancy (LE) among countries along B&R and studied the impact of economic development on LE. Data from 65 B&R countries from 2000 to 2014 were compiled and analyzed. Trend of LE was examined by sex and country. Linear quantile mixed model was used to study the associations between LE and economic factors. In 2014, the average LE in all B&R countries was 69.7 years for men and 73.7 years for women. Across countries in 2014, LE for men ranged from 58.6 years in Afghanistan to 80.2 years in Israel. LE for women ranged from 61.3 years in Afghanistan to 85.9 in Singapore. GDP per capita was positively associated with longevity across B&R countries. The unemployment rate was positively associated with LE only for countries in the top LE quantiles. GDP growth rate and Inflation were negatively associated with LE for the countries in the bottom LE quantiles for men, not for women. LE increased substantially among B&R countries during 2000–2014. The influence of macroeconomic factors on LE was related to the distribution of LE.

## 1. Introduction

Life expectancy (LE) is a common health indicator worldwide and serves as an important measure in public health. It reflects the quality of social life and allows inequality levels and trends to be compared within and across societies [1,2,3]. The trend in the LE in humans during the past thousand years has been characterized by a slow, steady increase, despite regional and major global health crises [4,5].

A few studies have shown that national income and other socioeconomic factors play an important role on LE based on a global scale [6,7,8,9,10,11,12,13]. In addition, countries with lower socioeconomic status may have higher death rates, but the magnitude of inequality between groups of higher and lower socioeconomic status are much larger in some countries than in others [14].

In 2013, China launched the Belt and Road (B&R) Initiative to promote trade, infrastructure, and commercial associations across countries in Asia, Africa, and Europe [15,16]. What the trend is in LE and how economic change affects the health conditions in B&R countries is inconclusive. Most countries included in the B&R Initiative are developing countries, in which LE is more likely to be affected by socioeconomic status, with their socioeconomic status lying between the highest and lowest in the world [14]. In addition, with the advance of the B&R Initiative, the entire economic situation in this region will change accordingly. Thus, to inform planning for health and social services and pensions, assessing the LE and how this economic change affects the health conditions especially LE and which aspects of socioeconomic determinants play a significant impact on LE are of importance.

Although previous studies have provided valuable relationships between socioeconomic factors and LE, their results do not give a full picture of the relationship between explanatory and outcome variables [6,7,8,9,10,11,12,13]. There is almost non-existent literature that uses quantile regression to estimate the association between LE and socioeconomic factors. Those studies only describe the conditional mean of an outcome without describing the scale of its distribution and the classification also lead to a loss of information. The linear quantile mixed models we employed in this paper provide a complete image of the effects of covariates on LE by estimating the family of conditional quantile function, making linear quantile mixed models a natural choice for this analysis due to their robustness and lack of distribution hypothesis [17].

Our study therefore aims to (1) document the change of LE among the countries along the B&R, and (2) assess the impact of economic factors on different quantiles of LE. We hypothesized that LE would correlate with the macroeconomic factors such as the gross domestic product (GDP) per capita, growth rate, inflation and unemployment rate, all of which have significant impacts on the daily life of the population.

## 2. Materials and Methods

### 2.1. Data Sources

Data on 65 B&R countries were collected from the World Bank database and World Health Organization database [18,19].

LE indicated the number of years a newborn infant would live if the prevailing patterns of mortality at the time of birth were to stay. LE in some years did not follow a normal distribution (Table S1) and skewness (Figure S1) in B&R countries. Total LE of B&R countries and total LE in the same year was weighted by the population size. Inflation, unemployment rate, GDP growth rate and GDP per capita were selected as economic factors, which are aspects of the economic situation that have a direct impact on the everyday life of the population and especially of vulnerable groups [20]. (1) GDP was the sum of gross value added by all resident producers in the economy, plus any product taxes, and minus any subsidies not included in the value of the products. It was calculated without making deductions for depreciation of fabricated assets, or for depletion and degradation of natural resources. (2) Inflation was measured by the annual growth rate of the GDP implicit deflator and showed the rate of price change in the economy as a whole. (3) Unemployment rate was defined as the share of the labor force that was without work, but available for and seeking employment. (4) GDP growth rate was defined as annual percentage growth rate of GDP at market prices based on constant local currency. Aggregates were based on 2010 U.S. dollars, calculated without making deductions for depreciation of fabricated assets, or for depletion and degradation of natural resources. To show the secular trend in life expectancy, we chose the years from 2000 to 2014 for comparison, which is the longest year difference, and this time frame provided a sufficient volume of data for statistical analysis described below.

### 2.2. Statistical Analysis

We examined the trends of LE in the B&R countries from 2000 to 2014 by sex and country. LE in the same year was obtained by population weighting. A linear quantile mixed model was used to allow for differential trajectories of LE across countries [17,21]. Nine quantiles of LE were selected (from 10th to 90th in increments of 10) from the lowest to the highest LE in the B&R countries. A random effect model was used to provide a basis for comparison with the linear quantile mixed model. The 95% confidence intervals were obtained using 1,000 bootstrap replications.

The dependent variable was LE. Using country as a categorical variable, and the economic variables as continuous predictors. GDP per capita was divided by 1000 to facilitate result interpretation. Countries with more than a year of missing data (i.e., Afghanistan, Syria, Qatar, Montenegro, Maldives, Iraq, Palestine) were excluded from the regression analysis.

Linear quantile mixed models provide a well-suited modeling framework for uncovering trends in multivariate data. Their main advantage over simple regression models is that they extend regression for modeling the mean to analyzing the entire conditional distribution of the outcome variable [17]. In addition, quantile regression does not require any assumptions about the distribution of the regression residuals and, unlike ordinary linear regression, is not influenced by outliers or skewness in the distribution of the dependent variable, providing greater statistical efficiency when outliers are present [22]. Therefore, location, scale and shape of the distribution can be examined through the analysis of conditional quantile models to provide a complete picture of the distributional effects.

All statistical analyses were performed using R 3.3.2. (R Foundation for Statistical Computing, Vienna, Austria). The linear quantile mixed model was developed using the package ‘lqmm’, and the random effect model using the package ‘plm’. Two-tailed *p*-value < 0.05 was considered statistically significant.

## 3. Results

### 3.1. Change of LE in the B&R Countries from 2000 to 2014

The average LE in the B&R countries increased from 2000 to 2014. In 2014, the LE in all the B&R countries was 69.7 years for men and 73.7 years for women, compared to 65.7 years and 69.6 years for men and women, respectively, in 2000. LE had risen by 4.0 years for men and 4.1 years for women from 2000 to 2014 (Table 1).

Across countries, LE for men ranged from 58.6 years in Afghanistan to 80.2 in Israel in 2014. LE for women ranged from 61.3 years in Afghanistan to 85.9 in Singapore in 2014. LE for men in 2014 was below 65 years for 9 countries compared with only Afghanistan for women’s LE. 12 countries had LE for women in 2014 of at least 80 years, but only Israel had LE for men of 80 years or higher (Table 2).

Changes in LE for men between 2000 and 2014 ranged from a decrease of 10.6 years in Syria, to an increase of 10.8 years in Cambodia. After Syria, the other decrease was in Iraq, which was 2.9 years. Changes in LE for women between 2000 and 2014 ranged from a decrease of 5.2 years in Syria to an increase of 10.3 years in Cambodia. LE for men increased by 5 years or more in 16 countries, compared with 14 countries for women LE (Table 2).

### 3.2. Associations between LE and Macroeconomic Factors

**Figure 1:**
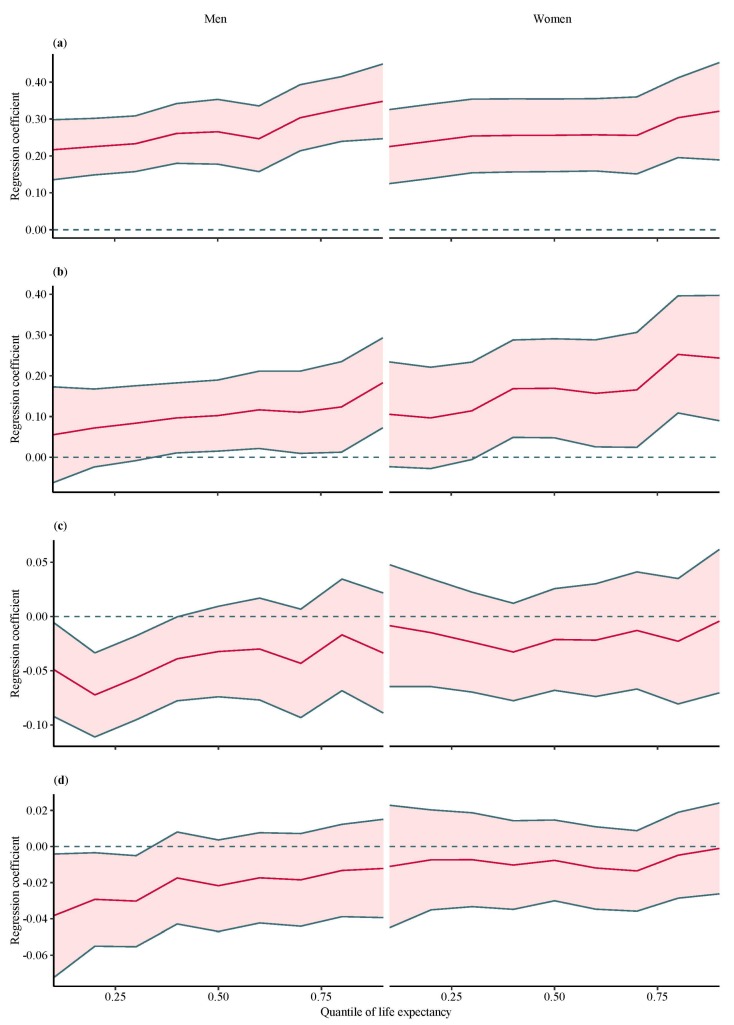
Quantile regression coefficients for life expectancy and macroeconomic factors on percentiles of total life expectancy in 65 Belt and Road countries. This picture shows the quantile regression coefficients (red line) with their 95% CI for the relationship between life expectancy and macroeconomic factors. (**a**) Quantile regression coefficients for GDP per capita and life expectancy on percentiles of total life expectancy; (**b**) Quantile regression coefficients for unemployment rate and life expectancy on percentiles of total life expectancy; (**c**) Quantile regression coefficients for GDP growth rate and life expectancy on percentiles of total life expectancy; (**d**) Quantile regression coefficients for inflation and life expectancy on percentiles of total life expectancy.

The results of the quantile regression analyses suggested that the associations between LE and economic factors were inconsistent across quantiles (Table 3). Figure 1 shows the quantile regression coefficients (solid line) with their 95% CI for the association between LE and four economic factors by gender.

The random effect model showed that, on average, GDP per capita had a positive effect on LE for both men and women. However, the random effect model did not give a complete picture of the association between the LE and GDP per capita. When we used the linear quantile mixed model, GDP per capita was positively associated with LE at all percentiles of LE distribution. All these regression coefficients showed a slightly increasing trend (from low to high quantiles of LE distribution), while the positive association between LE and GDP per capita was stronger in higher quantiles of LE distribution (e.g., 10th percentile estimate of women = 0.23, 95% CI: 0.13–0.33, 90th percentile estimate of women = 0.32, 95% CI: 0.19–0.45) (Figure 1a).

For unemployment rate, random effect model showed that there was no significant association between unemployment rate and LE for both men and women. Nevertheless, there was statistical significance analyzed by the linear quantile mixed model. Unemployment rate was positively associated with men’s and women’s LE for part of the quantiles (e.g., quantile ≥ 40th), with an increasing trend (Figure 1b).

The linear quantile mixed model showed that GDP growth rate was negatively associated with LE for men only for part of quantiles (≤40th) and showed a clear increasing trend (Figure 1c). Similar to GDP growth rate, inflation presented negative association with LE for men only for low quantiles (quantile ≤ 40th) with an increasing tendency (Figure 1d). While the linear quantile mixed model suggested no association of GDP growth rate and inflation on LE for women, the estimates from the random effect model suggested a negative association between them (Table 3).

## 4. Discussion

This study examined LE and its related influencing economic factors among the B&R countries from 2000 to 2014. LE was increasing in B&R countries, but the magnitude of increase varied across the B&R countries. The influence of economic factors (i.e., inflation, unemployment rate, GDP per capita, and annual percentage growth rate of GDP) on LE is related to the distribution of LE.

Gains in LE during 2000–2014 may be partly due to public health and health-care successes in these countries. Women in Singapore had the highest LE. With the effort to create a system of affordable health care for all Singaporeans, promoting aged health, controlling the main killer diseases, concentrating on children’s health, and managing mental health, today, Singapore is becoming a country with one of the highest LEs in the world [23]. Singapore also ranked among countries with the best health outcomes [24]. Overall, LE has risen in 63 of 65 countries. The two exceptions are Iraq and Syria, where the prolonged war in the first decade of the 21st century has erased years of LE at the population level [25,26], and up to 2014, the decline in LE had not been reversed. The failure to make substantial progress in increasing LE over the last 15 years should serve as a wake-up call to these countries.

Country-level determinants of LE have been extensively studied. Among these, national income was certainly the most widely acknowledged [13,14,27,28]. Our findings, consistent with previous studies, indicate that LE was positively associated with GDP per capita among both men and women. As the economy grows, there are more resources to improve nutrition, access medical care, and provide other conditions that contribute to the improvement of health and well-being. Furthermore, improvements in GDP per capita had different impact on the B&R countries, as the countries with higher LE were more affected by changes in GDP per capita. Our analyses of the B&R countries showed that higher income is associated with greater longevity throughout the income distribution [13]. However, this finding should be interpreted with caution, because some countries with a long life expectancy were economically underdeveloped, such as Costa Rica and Okinawa [29,30]. Interestingly, it seemed that there was a threshold in promoting LE by GDP per capita; even though increased GDP per capita can promote LE, beyond this threshold, LE will increase more.

Unemployment was an important social determinant of health and was strongly correlated with health outcomes [13]. Findings from this study indicated that there was a positive association between LE and unemployment in countries with higher LE in both men and women. The same conclusion had appeared in Europe, which can be partially explained by the “healthier immigrant effect” [31]. This effect refers to the self-selection process in which immigrants were healthier than native population [31]. Most countries with higher LE along B&R were located in the Eastern Europe region, which belong to middle- and high-income regions. As unemployment rates increased, middle- and high-income regions may have received immigrants from less developed countries [31]. Even though we identified a positive association between LE and unemployment rate, except for the “healthier immigrant effect”, reducing the unemployment rate may be an appropriate strategy to increase LE [32]. An inverse relationship between LE and unemployment has been consistently documented [31,33,34]. Compared to those in low-unemployment areas, people in higher-unemployment areas have higher prevalence of self-assessed fair/poor health, psychological distress, disability, functional limitation, and chronic conditions that impact on LE [34,35]. Effective policy strategies to reduce the health consequences of unemployment may include integration of employment and skill services, investment in job training and skills development, raising the minimum wage [34].

Our study indicated that men in countries with lower LE were more likely to be negatively affected by GDP growth rates, and with the increase of LE, the effect gradually decreased and finally disappeared. This negative association occurred in some industrialized countries many years ago [36], but for countries with lower LE along the B&R, this association may just be beginning, and was only evident in men. The countries with lower LE along B&R are developing countries, which have limited health care resources. Making the best use of resources is vital in these countries, which are struggling to improve public health with limited funds. Although, in our study, economic growth may have harmful effects on LE, higher mortality during temporary expansions may not imply negative effects of permanent economic growth. In the long term, economic growth contributes to better health [37]. On the premise of economic growth, advocating for healthy lifestyle and better workplace environment, especially for men in countries with lower LE, may be an effective way to promote health and increase LE.

A previous study reported that inflation seemed to have a significant negative impact on gains in longevity many years ago in Sweden [36], but now, this association is occurring for countries with lower LE along the B&R in men. On the one hand, people’s expenditures are increased by any growth in inflation, which in turn leads to a reduction of the share of various sectors, such as hygiene and medicine. This reduction means less medical care and harms the health. On the other hand, the psychological effects of inflation have a destructive influence on the people, as well as a negative impact on their behavior and morality. Though increasing the inflation rate seems harmful to men in our study, it does not mean that the lowest inflation rate is good for health. Pursuing lower inflation may interfere with other economic targets, including reduction of unemployment or avoiding drops in GDP [38], which impact LE. From a public health perspective, reducing the inflation rate to an appropriate level seems a better method to maintain population health, particularly in countries with lower LE. Our study had several strengths. The primary strength of the present study is that it is the first to examine LE among the B&R countries as a whole, and the first study to report the impact of socioeconomic factors on LE among the B&R countries. Importantly, our study used linear quantile mixed models, a semi-parametric robust regression technique that allows the LE to be treated continuously. It has several advantages that apply directly to the analysis of our data set. Firstly, the research is based on quantiles, rather than on the means of LE. Our study interest was the complete distribution of LE. Inference based on mean LE alone would not be as informative as inference based on multiple quantiles throughout the distribution. Secondly, quantile regression has statistical efficiency and robustness to outliers. Outlying and large values have an important effect on the mean, and therefore have an impact on linear regression estimates. However, quantile regression is robust to them. Thirdly, quantile regression does not make any request of the distribution of the regression residuals, and is not influenced by outliers or skewness in the distribution of the dependent variable, providing greater statistical efficiency when outliers are present [39].

Findings were inconsistent between linear quantile mixed models and random effect models. This was because the random effect model focuses on the conditional distribution of the outcome only through its mean. Some of our findings suggested that the distribution of LE for women was not normal and was quite skewed; thus, focusing on average LE may not be suitable. The violation of the normality assumption could explain some of the inconsistencies between the two approaches.

This study has several limitations. First, we used an ecological design that does not allow us to interpret the result at the individual level. Second, the data was collected from national statistical agencies, and there may be differences in data quality among countries, which could lead to potential bias in estimating LE. Third, the study data timeframe we focused on was relatively short, only from 2000 to 2014. If more data could be collected over a longer term, better understanding of the changing trends of LE and the correlation between LE and economic variables could be achieved.

## 5. Conclusions

In summary, this study described the changes in LE and assessed the associations between LE and macroeconomic factors in 65 the B&R countries. It showed that the influence of economic factors on LE was related to the distribution of LE. LE in higher LE countries were more vulnerable to unemployment rate for both men and women. GDP per capita was positively associated with greater longevity throughout the LE distribution. LE for men in lower LE countries was more vulnerable to inflation and GDP growth rate. Although the relationship between macroeconomic factors and LE seems complex, economic development will benefit public health in the long run.

## Figures and Tables

**Table 1:** Change of life expectancy in all 65 Belt and Road countries, 2000–2014.

Year	Women	Men
2000	69.6	65.7
2001	69.9	66.1
2002	70.2	66.4
2003	70.5	66.7
2004	70.8	67.0
2005	71.1	67.3
2006	71.4	67.6
2007	71.8	67.9
2008	72.0	68.2
2009	72.3	68.5
2010	72.6	68.7
2011	72.9	69.0
2012	73.2	69.2
2013	73.4	69.5
2014	73.7	69.7

Total life expectancy in the same year was weighted by the population size.

**Table 2:** Change of life expectancy years in 65 Belt and Road countries, 2000–2014.

Country	2000		2014		15 Years Increase in Life Expectancy	
	Women	Men	Women	Men	Women	Men
Afghanistan	56.1	53.7	61.3	58.6	5.2	4.9
Albania	76.4	69.4	80.6	74.7	4.2	5.3
Armenia	75.1	68.7	77.5	71.4	2.4	2.7
Azerbaijan	69.8	63.4	75.5	69.4	5.7	6.0
Bahrain	75.5	73.7	77.8	76.0	2.3	2.3
Bangladesh	65.6	64.9	72.7	70.2	7.1	5.3
Belarus	73.5	62.6	77.7	66.3	4.2	3.7
Bhutan	60.3	60.0	69.7	69.2	9.4	9.2
Bosnia and Herzegovina	77.2	71.8	79.6	74.8	2.4	3.0
Brunei Darussalam	76.0	73.1	79.1	76.2	3.1	3.1
Bulgaria	74.7	67.8	77.8	70.9	3.1	3.1
Cambodia	59.9	55.3	70.2	66.1	10.3	10.8
China	73.5	70.1	77.4	74.4	3.9	4.3
Croatia	78.2	71.1	81.0	74.4	2.8	3.3
Cyprus	80.2	76.0	82.5	78.2	2.3	2.2
Czech Republic	78.1	71.2	81.5	75.6	3.4	4.4
Egypt	71.4	66.4	73.0	68.6	1.6	2.2
Estonia	76.1	65.4	81.8	72.3	5.7	6.9
Georgia	75.5	68.0	78.4	70.5	2.9	2.5
Greece	81.1	75.4	83.5	78.1	2.4	2.7
Hungary	76.0	67.5	79.0	72.1	3.0	4.6
India	63.4	61.7	69.5	66.6	6.1	4.9
Indonesia	68.0	64.6	71.0	66.9	3.0	2.3
Iran	71.2	69.4	76.5	74.4	5.3	5.0
Iraq	72.3	67.8	71.1	64.9	−1.2	−2.9
Israel	80.8	76.8	84.0	80.2	3.2	3.4
Jordan	73.3	70.4	75.7	72.4	2.4	2.0
Kazakhstan	69.9	58.3	74.5	65.3	4.6	7.0
Kuwait	74.4	72.4	75.8	73.6	1.4	1.2
Kyrgyzstan	70.7	62.6	74.8	66.9	4.1	4.3
Laos	59.7	56.5	66.8	63.7	7.1	7.2
Latvia	75.7	64.4	79.0	69.4	3.3	5.0
Lebanon	74.3	71.2	76.4	73.4	2.1	2.2
Lithuania	77.2	65.9	79.0	67.9	1.8	2.0
Macedonia	74.9	70.5	77.7	73.4	2.8	2.9
Malaysia	74.9	70.2	77.1	72.5	2.2	2.3
Maldives	70.4	68.9	79.8	76.6	9.4	7.7
Mongolia	65.7	60.0	72.8	64.3	7.1	4.3
Montenegro	75.6	70.4	77.9	74.0	2.3	3.6
Myanmar	64.1	60.2	68.3	64.4	4.2	4.2
Nepal	63.5	61.5	71.0	68.2	7.5	6.7
Oman	74.9	70.7	79.0	74.7	4.1	4.0
Pakistan	63.6	62.0	67.2	65.2	3.6	3.2
Philippines	69.9	63.9	71.8	65.2	1.9	1.3
Poland	77.9	69.5	81.2	73.4	3.3	3.9
Qatar	77.8	75.3	79.9	77.3	2.1	2.0
Republic of Moldova	71.0	63.2	75.9	67.6	4.9	4.4
Romania	74.3	67.2	78.6	71.2	4.3	4.0
Russian Federation	72.0	58.7	76.1	64.5	4.1	5.8
Saudi Arabia	74.4	71.0	75.9	73.1	1.5	2.1
Serbia	75.6	69.7	78.2	72.7	2.6	3.0
Singapore	80.9	75.9	85.9	79.8	5.0	3.9
Slovakia	77.1	68.9	80.1	72.7	3.0	3.8
Slovenia	79.7	72.2	83.6	77.7	3.9	5.5
Sri Lanka	75.1	68.2	78.1	71.3	3.0	3.1
Syrian Arab Republic	74.9	70.5	69.7	59.9	-5.2	-10.6
Tajikistan	67.6	60.3	73.4	66.4	5.8	6.1
Thailand	74.6	67.8	77.8	71.7	3.2	3.9
Turkey	73.8	67.1	78.6	72.3	4.8	5.2
Turkmenistan	67.9	59.9	70.3	62.0	2.4	2.1
Ukraine	73.2	62.0	75.8	65.7	2.6	3.7
United Arab Emirates	75.7	73.4	78.5	76.2	2.8	2.8
Uzbekistan	70.4	63.8	72.6	65.9	2.2	2.1
Viet Nam	78.2	68.5	80.5	71.1	2.3	2.6
Yemen	62.2	59.5	66.9	63.9	4.7	4.4
Range	56.1–81.1	53.7–76.8	61.3–85.9	58.6–80.2	−5.2–10.3	−10.6–10.8
Total	69.6	65.7	73.7	69.7	4.1	4.0

Total life expectancy of Belt and Road countries was weighted by the population size.

**Table 3:** Association between life expectancy and macroeconomic factors in 65 Belt and Road countries: linear quantile mixed model.

		10th Percentile ^1^	20th Percentile ^1^	30th Percentile ^1^	40th Percentile ^1^	50th Percentile ^1^	60th Percentile ^1^	70th Percentile ^1^	80th Percentile ^1^	90th Percentile ^1^	Random Effect Model ^2^
	1. Women										
GDP growth rate (%)	Coefficient ^3^	−0.01	−0.02	−0.02	−0.03	−0.02	−0.02	−0.01	−0.02	0.00	−0.04
	95% CI ^4^	(−0.07, 0.05)	(−0.07, 0.04)	(−0.07, 0.02)	(−0.08, 0.01)	(−0.07, 0.03)	(−0.07, 0.03)	(−0.07, 0.04)	(−0.08, 0.04)	(−0.07, 0.06)	(−0.06, −0.02)
	*p*-value	0.768	0.557	0.314	0.153	0.376	0.411	0.641	0.439	0.899	<0.001
Unemployment rate (%)	Coefficient ^3^	0.11	0.10	0.11	0.17	0.17	0.16	0.17	0.25	0.24	0.00
	95% CI ^4^	(−0.02, 0.23)	(−0.03, 0.22)	(−0.01, 0.23)	(0.05, 0.29)	(0.05, 0.29)	(0.03, 0.29)	(0.02, 0.31)	(0.11, 0.40)	(0.09, 0.40)	(−0.04, 0.04)
	*p*-value	0.108	0.128	0.062	0.006	0.006	0.019	0.022	0.001	0.002	0.965
GDP per capita/1000	Coefficient ^3^	0.23	0.24	0.25	0.26	0.26	0.26	0.26	0.30	0.32	0.17
	95% CI ^4^	(0.13, 0.33	(0.14, 0.34)	(0.15, 0.35)	(0.16, 0.36)	(0.16, 0.35)	(0.16, 0.36)	(0.15, 0.36)	(0.20, 0.41)	(0.19,0.45)	(0.15, 0.19)
	*p*-value	<0.001	<0.001	<0.001	<0.001	<0.001	<0.001	<0.001	<0.001	<0.001	<0.001
Inflation (%)	Coefficient ^3^	−0.01	−0.01	-0.01	−0.01	−0.01	−0.01	−0.01	−0.01	0.00	−0.02
	95% CI ^4^	(−0.05, 0.02)	(−0.04, 0.02)	(−0.03, 0.02)	(−0.04, 0.01)	(−0.03, 0.02)	(−0.04, 0.01)	(−0.04, 0.01)	(−0.03, 0.02)	(−0.03,0.02)	(−0.03, −0.01)
	*p*-value	0.522	0.600	0.581	0.412	0.500	0.306	0.234	0.690	0.933	<0.001
	2. Men										
GDP growth rate (%)	Coefficient ^3^	−0.05	−0.07	−0.06	−0.04	−0.03	−0.03	−0.04	−0.02	−0.03	−0.06
	95% CI ^4^	(−0.09, −0.01)	(−0.11, −0.03)	(−0.10, −0.02)	(−0.08, 0.00)	(−0.07, 0.01)	(−0.08, 0.02)	(−0.09, 0.01)	(−0.07, 0.04)	(−0.09,0.02)	(−0.08, −0.04)
	*p*-value	0.027	0.000	0.004	0.048	0.129	0.211	0.090	0.519	0.234	<0.001
Unemployment rate (%)	Coefficient ^3^	0.06	0.07	0.08	0.10	0.10	0.12	0.11	0.12	0.18	0.01
	95% CI ^4^	(−0.06, 0.17)	(−0.02, 0.17)	(−0.01, 0.18)	(0.01, 0.18)	(0.02, 0.19)	(0.02, 0.21)	(0.01, 0.21)	(0.01, 0.24)	(0.07,0.29)	(−0.03, 0.04)
	*p*-value	0.360	0.142	0.076	0.028	0.022	0.016	0.032	0.030	0.001	0.811
GDP per capita/1000	Coefficient ^3^	0.22	0.23	0.23	0.26	0.27	0.25	0.30	0.33	0.35	0.19
	95% CI ^4^	(0.14, 0.30)	(0.15, 0.30)	(0.16, 0.31)	(0.18, 0.34)	(0.18, 0.35)	(0.16, 0.34)	(0.21, 0.39)	(0.24, 0.42)	(0.25,0.45)	(0.17, 0.21)
	*p*-value	<0.001	<0.001	<0.001	<0.001	<0.001	<0.001	<0.001	<0.001	<0.001	<0.001
Inflation (%)	Coefficient ^3^	−0.04	−0.03	−0.03	−0.02	−0.02	−0.02	−0.02	−0.01	−0.01	−0.02
	95% CI ^4^	(−0.07, 0.00)	(−0.06, 0.00)	(−0.06, −0.01)	(−0.04, 0.01)	(−0.05, 0.00)	(−0.04, 0.01)	(−0.04, 0.01)	(−0.04, 0.01)	(−0.04,0.02)	(−0.03, −0.01)
	*p*-value	0.028	0.026	0.019	0.179	0.093	0.173	0.158	0.308	0.381	<0.001

^1^ Linear quantile mixed model, the dependent variables were nine percentiles (10th, 20th, 30th, 40th, 50th, 60th, 70th, 80th, 90th) of the distribution of life expectancy for women and men. Country was used as a classification prediction and the economic variables (inflation, unemployment rate, GDP per capita, annual percentage growth rate of GDP) as continuous predictors. ^2^ Random effect model, the dependent variable was life expectancy for women and men. Country was used as a classification prediction and the economic variables as continuous predictors. ^3^ The coefficient represents the change in the value at the *n*th percentile of life expectancy unit change in the independent variable. For interactions, the coefficient was the difference in the change in the value of life expectancy at the *n*th percentile compared to the main relative to the change when the interacting variable was at its reference level. ^4^ Linear quantile mixed model’s confidence intervals (CI) were obtained using 1000 bootstrap replications.

## Supplementary Materials

The following are available online at http://www.mdpi.com/1660-4601/15/12/2890/s1, Figure S1: Probability density of life expectancy in B&R countries, Table S1: Normal distribution test of life expectancy by year.
