# Supplementary material for: Trends in Life Expectancy and Its Association with Economic Factors in the Belt and Road Countries—Evidence from 2000–2014
Source: Int J Environ Res Public Health. 2018 Dec 17;15(12):2890. doi: 10.3390/ijerph15122890 (PMC6313698; doi:10.3390/ijerph15122890)
Supplement: Supplementary file 1 [file ijerph-15-02890-s001.pdf]

Supplements document

Table S1. Normal distribution test of life expectancy by year.

Year	Sex	Shapiro-Wilk statistic	*p*-value
2000	Women	0.924	0.001
	Men	0.978	0.350
2001	Women	0.932	0.003
	Men	0.973	0.206
2002	Women	0.941	0.007
	Men	0.971	0.173
2003	Women	0.944	0.009
	Men	0.972	0.192
2004	Women	0.951	0.018
	Men	0.970	0.152
2005	Women	0.951	0.018
	Men	0.964	0.082
2006	Women	0.958	0.038
	Men	0.967	0.106
2007	Women	0.960	0.051
	Men	0.967	0.113
2008	Women	0.963	0.073
	Men	0.983	0.570
2009	Women	0.967	0.108
	Men	0.966	0.100
2010	Women	0.969	0.135
	Men	0.964	0.074
2011	Women	0.968	0.127
	Men	0.968	0.128
2012	Women	0.967	0.108
	Men	0.974	0.235
2013	Women	0.968	0.116
	Men	0.977	0.327
2014	Women	0.970	0.159
	Men	0.974	0.242

Normal distribution test for belt and road country's life expectancy. From 2000 to 2006, through Shapiro-Wilk test, women's life expectancy was not normal.

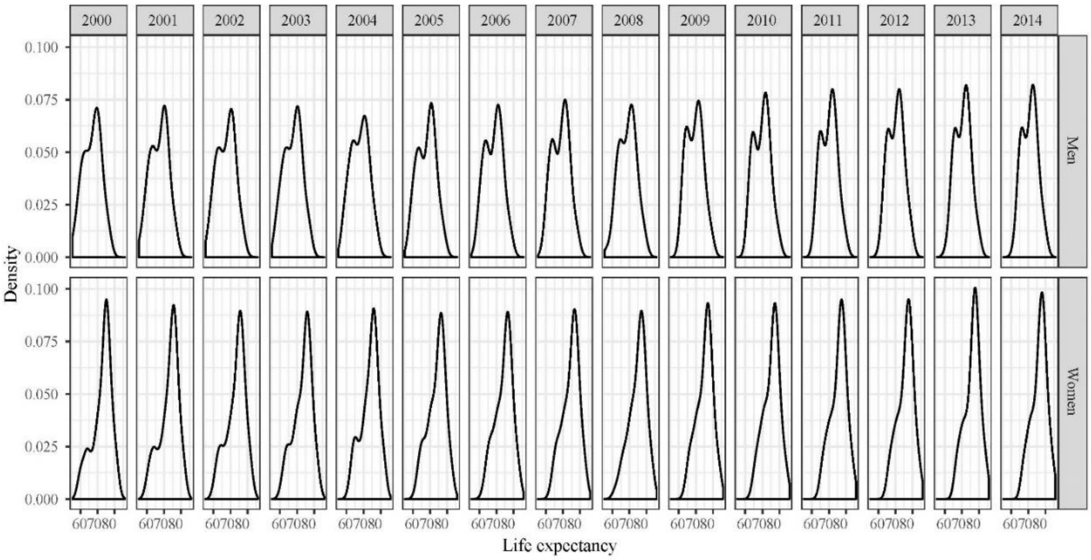

Figure S1. Probability density of life expectancy in B&R countries.
